# Effects of Perch Essence Supplementation on Improving Exercise Performance and Anti-Fatigue in Mice

**DOI:** 10.3390/ijerph19031155

**Published:** 2022-01-20

**Authors:** Mon-Chien Lee, Yi-Ju Hsu, Yu-Qian Lin, Ling-Ni Chen, Mu-Tsung Chen, Chi-Chang Huang

**Affiliations:** 1 Graduate Institute of Sports Science, National Taiwan Sport University, Taoyuan City 333, Taiwan; 1061304@ntsu.edu.tw (M.-C.L.); ruby780202@ntsu.edu.tw (Y.-J.H.); 1090204@ntsu.edu.tw (Y.-Q.L.); 2 Anyong Biotechnology, Inc., Kaohsiung City 827, Taiwan; celia.chen@topco-global.com; 3 Department of Food and Beverage Management, Shih Chien University, Taipei City 104, Taiwan; bruce@g2.usc.edu.tw

**Keywords:** exercise performance, perch essence, anti-fatigue, fish soup

## Abstract

Silver perch (*Bidyanus bidyanus*) has many nutrition and health benefits, being a rich source of macro and micronutrients, phospholipids, polyunsaturated fatty acids, and a variety of essential minerals while having a high protein content. In addition to direct consumption, it is often made into a soup as an important nutritional supplement for strengthening the body and delaying fatigue. By extracting the essence, its quality can be controlled, and it is convenient to supplement. This study aimed to evaluate the effect of supplementation with Santé premium silver perch essence (SPSPE) on improving exercise performance and anti-fatigue. Fifty male institute of cancer research (ICR) mice were divided into five groups (*n* = 10/group): (1) vehicle (vehicle control or water only), (2) isocaloric (0.93 g casein/kg/mice/day), (3) SPSPE-1X (0.99 g/kg/mice/day), (4) SPSPE-2X (1.98 g/kg/mice/day), and (5) SPSPE-5X (4.95 g/kg/mice/day). A sample or an equal volume of liquid was fed orally for four consecutive weeks. Grip strength and swimming exhaustion tests were used as exercise performance assessments. After 10 and 90 min of unloaded swimming, biochemical parameters of fatigue were evaluated. We found that supplementation with SPSPE for four consecutive weeks could significantly improve mice’s grip strength, exercise endurance performance, and glycogen content (*p* < 0.05), and significantly reduced post-exercise fatigue biochemical parameters, such as lactate, blood ammonia (NH_3_), blood urea nitrogen (BUN) concentration, and muscle damage index creatine kinase (CK) activity (*p* < 0.05). In summary, supplementation with SPSPE for 4 weeks could effectively improve exercise performance, reduce sports fatigue, and accelerate fatigue recovery. In addition, it did not cause any physiological or histopathological damage.

## 1. Introduction

In the traditional East Asian food culture, pork bones, chicken, fish, Chinese medicinal materials, etc. are often used to make soups as important nutritional supplements for strengthening the body and delaying fatigue. Among them, fish is a rich source of macro and micronutrients, phospholipids, polyunsaturated fatty acids, and a variety of essential minerals while having a high protein content [1]. However, fish needs to be eaten as soon as possible to avoid biochemical and microbial decay, which results in toxic substances [2]. Therefore, fish protein hydrolysate or fish processed into ready-to-drink meat soup or essence soup are some the most effective methods used to preserve its nutritional value. Fish protein hydrolysate (FPH) mainly contains dipeptides and tripeptides, which are easier to absorb than free amino acids and intact protein [3]. In addition, compared with the same weight of whey protein hydrolysate, it has a higher total antioxidant capacity [4]. Fish protein-derived bioactive peptides can improve endothelial dysfunction through antihypertensive and antioxidant effects [5]. In animal experiments, FPH has been proven to be beneficial, improving cardiovascular, neurological, intestinal, renal, and immune health [6]. On the other hand, thermal processing helps to decompose the macromolecules in the meat into micron- or nano-sized particles, which can effectively delay the generation of free radicals in the body and related diseases [7].

Silver perch (*Bidyanus bidyanus*) is native to Australia. It was introduced into Taiwan for farming in the 1990s and is now commercially farmed. Silver perch is an excellent source of nutrition and health benefits for humans and is rich in unsaturated fatty acids (including omega-3 and omega-6), protein, and essential amino acids [8]. Long-term simmering of perch soup does not affect the free amino acids and essential amino acids but does improve the flavor and taste, and increase phenolics, Maillard reaction products (MRPs), and amino acids, which may help to enhance its antioxidant effects [8]. Therefore, sea perch essence is an excellent source of food rich in metabolically active and healthy ingredients. It is not only suitable for all ages, but also as a nutritional supplement for elderly, pregnant and postpartum women, and infirm and postoperative patients to delay fatigue and accelerate recovery.

Fatigue is mainly caused by adverse reactions due to physical and mental factors or disease [9]. Physiological factors often include physical overload, during which the original or expected functional performance cannot be maintained [10]. Roughly divided into central fatigue and peripheral fatigue, fatigue is a common and complex multidimensional symptom [11]. Peripheral fatigue generally refers to the inability of the body to provide or maintain the required energy load under long-term or high-intensity activities, resulting in performance degradation [12]. During exercise, various energy sources, such as liver glycogen and glucose, are metabolized and used [13]. When energy is depleted and the supply and demand are imbalanced, the by-products of fatigue will increase, including the accumulation of lactic acid, inorganic phosphorus, and ammonia. This makes it difficult for muscles to maintain continuous contraction, resulting in an inability to maintain or a loss of their original exercise performance [14]. Therefore, in addition to improving physical fitness through regular scientific training, the use of nutritional supplements to improve the utilization of energy in the body, reduce consumption, delay the accumulation of fatigue metabolites, and accelerate the elimination of fatigue is one effective strategy [15].

In recent years, protein-rich nutritional supplements have become a popular choice for improving exercise performance, including whey, isolate soy protein, etc. In addition, many studies have confirmed that chicken essence rich in branched-chain amino acids (BCAAs) significantly improved exercise performance and delayed sports fatigue [15,16]. However, few studies have investigated fish food or essence extracts. Therefore, the purpose of this study was to explore the benefits of Santé premium silver perch essence (SPSPE) (a supplement rich in protein and amino acids) in improving exercise endurance performance and delaying fatigue-related biochemical indicators after 4 consecutive weeks of supplementation. In addition, we examined whether 4 consecutive weeks of SPSPE supplementation resulted in physiological maladaptation via blood biochemistry and histopathology.

## 2. Materials and Methods

### 2.1. Materials

The recommended daily intake of SPSPE for an adult weighing 60 kg is 60 mL (1 mL/kg body weight), which was provided by the Anyong Biotechnology, Inc. (Kaohsiung City, Taiwan). In addition, in order to ensure accurate administration to animals, heat-sterilized SPSPE was freeze-dried to obtain a powder extract. After freeze-drying the actual 60 mL product, 4.85 g of freeze-dried solids were obtained (freeze-drying rate of 8.08%). SPSPE was confirmed by Intertek Testing Services Taiwan Ltd. (Taipei, Taiwan) and contained 90.8 g of protein and 6.35 g of BCAA in 100 g of SPSPE freeze-dried powder (Table 1). According to the US Food and Drug Administration, a conversion factor of 12.3 was used to account for the difference in the body surface area between mice and humans. Therefore, the daily dose for mice was 845 mg/kg. In this study, in addition to designing 1X, 2X, and 5X doses to compare the benefits of different doses, an isocaloric group was added to eliminate the effects of supplemental calories.

### 2.2. Animals, and Experiment Design

Six-week old male institute of cancer research (ICR) mice were obtained from BioLASCO Taiwan (Yi-Lan Breeding Center, Yi-Lan County, Taiwan). All animal experiments were approved by the Institutional Animal Care and Use Committee (IACUC) of National Taiwan Sport University (IACUC-11019). The mice were adapted to the environment before the experiment and provided food ad libitum for 2 weeks. All animals were provided a standard laboratory diet (No. 5001; PMI Nutrition International, Brentwood, MO, USA) and distilled water ad libitum, and maintained in a 12-h light/12-h dark cycle at room temperature (22 ± 2 °C) and 60–70% humidity. In total, 50 mice were randomly assigned to 5 groups (10 mice/group): (1) vehicle (vehicle control or water only), (2) isocaloric (0.93 g casein/kg/mice/day), (3) SPSPE-1X (0.99 g/kg/mice/day), (4) SPSPE-2X (1.98 g/kg/mice/day), and (5) SPSPE-5X (4.95 g/kg/mice/day). All mice received samples with SPSPE by oral gavage for four consecutive weeks, and body weight, water consumption, and food intake were recorded each week.

### 2.3. Grip Strength

A digital low-force testing system (Model-RX-5, Aikoh Engineering, Nagoya, Japan) with a tension rod (diameter 2 mm, length 7.5 cm) and a force sensor was used to measure the grip strength of mice. We measured each mouse 10 times continuously and recorded the maximum value, as previously described [17].

### 2.4. Swimming Exercise Endurance Test

As described in our previous study [18], a weight equivalent to 5% of the mouse’s body weight (BW) was loaded on the tail of the test mouse and placed individually in a columnar swimming pool (65 cm high with a 20 cm radius) that was filled with water to a depth of 40 cm and maintained at 27 ± 1 °C. The swimming endurance time of each mouse was recorded from beginning to exhaustion. The criterion of fatigue was loss of coordinated movement or failure to return to the surface within 7 s.

### 2.5. Determination of Fatigue-Associated Biochemical Variables

To assess the effect of supplemental SPSPE on fatigue-related indicators and physiological status after exercise, all mice were fasted for 12 h prior to the collection of blood samples at baseline, after swimming unloaded for 10 min, and after resting for 20 min to analyze lactate, blood ammonia (NH_3_), and glucose. In addition, after 90 min of prolonged exercise and 60 min of rest, we evaluated blood urine nitrogen (BUN) and creatine kinase (CK), as previously described [19]. The blood samples were centrifuged at 1500× *g* for 15 min at 4 °C to collect serum for analysis. All serum samples were measured with an automatic analyzer (model 717, Hitachi, Tokyo, Japan).

### 2.6. Clinical Biochemical Profiles

At the end of the experimental period, all mice were sacrificed with 95% CO_2_ asphyxiation, and blood was immediately collected. Serum was separated by centrifugation and clinical biochemical variables, including aspartate aminotransferase (AST), alanine transaminase (ALT), albumin (ALB), total protein (TP), BUN, creatinine (CREA), uric acid (UA), total cholesterol (TC), triglycerides (TGs), creatine kinase (CK), and glucose (GLU), were measured with the aforementioned autoanalyzer (Hitachi 717, Hitachi, 63Tokyo, Japan).

### 2.7. Serum Myoglobin

We used commercial kits to analyze serum myoglobin based on a sandwich enzyme immunoassay (Crystal Chem’s Mouse Myoglobin ELISA Kit, Item Number: 80654, Crystal Chem, Elk Grove Village, IL, USA). The myoglobin present in the sample binds with antibodies adsorbed in the surface of the microplate. After standing at room temperature for 1 h, and repeated washing with washing solution, 100 μL of enzyme conjugate were added to each well, left to stand for 30 min, and then washing was repeated. Then, 100 μL of substrate solution were added to each well, left to stand for 10 min, 100 μL of stop solution added, and measurement performed at the 450/630 nm wavelength.

### 2.8. Body Composition, Histopathology, and Glycogen Content

After the mice were euthanized, the liver, kidneys, heart, lungs, muscles, epididymal fat pad (EFP), and brown adipose tissue (BAT) of mice were excised and weighed. The tissues were carefully removed, chopped, and fixed in 10% formalin, and then embedded in paraffin and cut into 4-μm-thick sections for morphological and pathological evaluation. Tissue sections were stained with hematoxylin and eosin (H & E) and examined by a veterinary pathologist using an optical microscope equipped with a CCD camera (BX-51, Olympus, Tokyo, Japan). As previously described [17], parts of the muscle and liver tissues were stored in liquid nitrogen for glycogen content analysis.

### 2.9. Statistical Analysis

All data are expressed as mean ± SD for *n* = 10 mice per group. Statistical differences among groups were analyzed by a one-way analysis of variance (ANOVA). The Cochran–Armitage test was used for the dose–effect trend analysis with SAS ver. 9.0 (SAS Institute, Cary, NC, USA). *p* < 0.05 was considered statistically significant.

## 3. Results

### 3.1. General Characteristics of Mice after SPSPE Supplementation for Four Weeks

As shown in Table 2, after 4 consecutive weeks of supplementation with SPSPE, there was no significant difference in the food, total calorie, and water intake of the mice in each group, and the body weight of the mice in each group showed a steady increasing trend every week. In addition, there was no significant difference in the absolute or relative (the relative organ weight was calculated by dividing the absolute weight by the mouse weight and converting it to a percentage) weight of the tissues and organs of the mice in each group.

### 3.2. Effect of SPSPE Supplementation on Grip Strength

**Figure 1:**
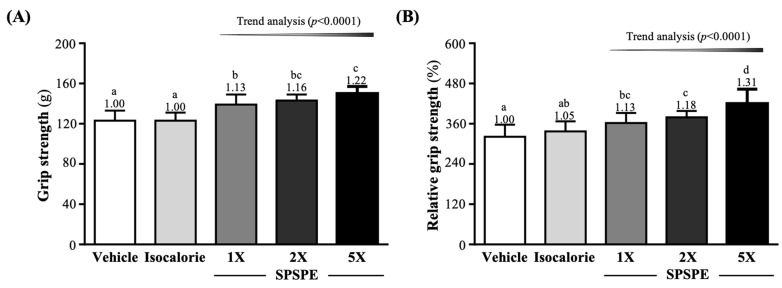
Effect of supplementation with SPSPE on (**A**) absolute forelimb grip strength and (**B**) forelimb grip strength (%) relative to body weight. Data are expressed as mean ± SD (*n* = 10 mice per group). The different superscript letters (a, b, c, d) above each bar indicate significant differences between the groups (*p* < 0.05).

The forelimb grip strength values in the vehicle, isocaloric, SPSPE-1X, SPSPE-2X, and SPSPE-5X groups were 123 ± 10, 123 ± 8, 139 ± 10, 143 ± 6, and 150 ± 7 g, respectively. Compared with the vehicle and isocaloric group, the SPSPE-1X, SPSPE-2X, and SPSPE-5X groups were significantly greater by 1.13-fold (*p* < 0.0001), 1.16-fold (*p* < 0.0001), and 1.22-fold (*p* < 0.0001), respectively (Figure 1A). The relative grip strength (%), normalized to body weight, of the SPSPE-1X, SPSPE-2X, and SPSPE-5X groups was significantly greater than the vehicle group by 1.13-fold (*p* = 0.0075), 1.18-fold (*p* = 0.0003), and 1.31-fold (*p* < 0.0001), respectively. However, compared with the isocaloric group, the SPSPE-2X and SPSPE-5X groups were significantly greater by 1.12-fold (*p* < 0.0066) and 1.25-fold (*p* < 0.0001), respectively (Figure 1B). The effect of supplementation with SPSPE on the absolute and relative grip strength was dose dependent (trend analysis, *p* < 0.0001).

### 3.3. Effect of SPSPE Supplementation in the Exhaustive Swimming Test

**Figure 2:**
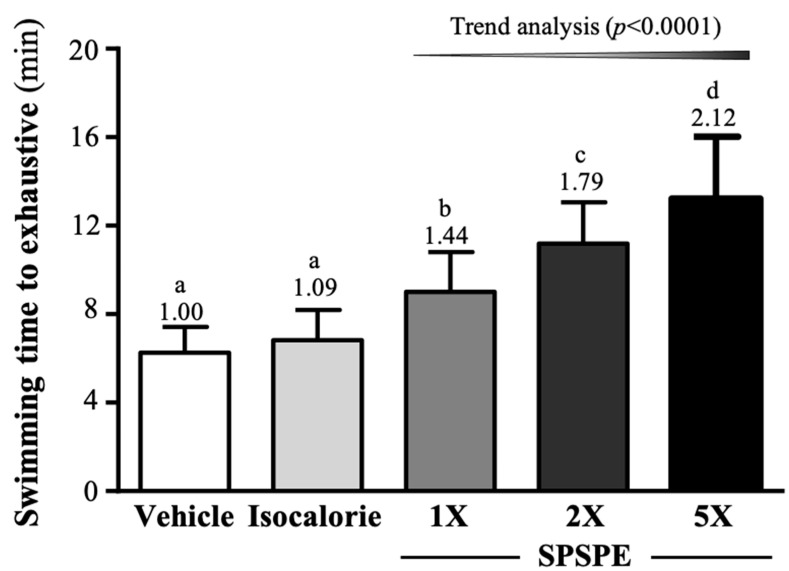
Effect of supplementation with SPSPE on exhaustive swimming time in mice. Data are expressed as mean ± SD (*n* = 10 mice per group). The different superscript letters (a, b, c, d) above each bar indicate a significant difference between the groups (*p* < 0.05).

The exhaustive swimming time in the vehicle, isocaloric, SPSPE-1X, SPSPE-2X, and SPSPE-5X groups was 6.25 ± 1.18, 6.83 ± 1.37, 9.00 ± 1.81, 11.19 ± 1.87, and 13.22 ± 2.80 min. Compared with the vehicle group, the SPSPE-1X, SPSPE-2X, and SPSPE-5X groups were significantly greater by 1.44-fold (*p* = 0.0021), 1.79-fold (*p* < 0.0001), and 2.12-fold (*p* < 0.0001), respectively. In addition, compared with the isocaloric group, the SPSPE-1X, SPSPE-2X, and SPSPE-5X groups were significantly greater by 1.32-fold (*p* = 0.00136), 1.64-fold (*p* < 0.0001), and 1.94-fold (*p* < 0.0001), respectively (Figure 2). The enhancement in the swimming capacity and endurance was shown to be dose dependent (*p* < 0.0001).

### 3.4. Effect of SPSPE Supplementation on Serum Lactate Levels after the 10-min Swim Test

We used 10 min of unloaded swimming to evaluate changes in the blood lactate levels in mice after 4 consecutive weeks of SPSPE supplementation. There was no significant difference between groups before swimming. After 10 min of swimming, the SPSPE-1X, SPSPE-2X, and SPSPE-5X groups’ levels were significantly lower than the vehicle group (by 19.19% (*p* < 0.0001), 21.56% (*p* < 0.0001), and 27.59% (*p* < 0.0001), respectively) and isocaloric group (by 14.86% (*p* = 0.0006), 17.36% (*p* < 0.0001), and 23.71% (*p* < 0.0001), respectively). The lactate production rate was calculated from the lactate level before exercise and 10 min after exercise. Compared with the vehicle group, the isocaloric, SPSPE-1X, SPSPE-2X, and SPSPE-5X groups’ levels were significantly reduced by 4.68% (*p* = 0.0001), 17.37% (*p* < 0.0001), 24.57% (*p* < 0.0001), and 27.81% (*p* < 0.0001), respectively. In addition, the SPSPE-1X, SPSPE-2X, and SPSPE-5X groups’ levels were also significantly reduced by 13.31% (*p* < 0.0001), 20.87% (*p* < 0.0001), and 24.27% (*p* < 0.0001), respectively, compared to the isocaloric group.

After 20 min of resting following the swimming test, the SPSPE-1X, SPSPE-2X, and SPSPE-5X groups’ levels were significantly lower than the vehicle group (by 23.01%, 25.63%, and 33.17%, respectively) and the isocaloric group (by 19.10%, 21.86%, and 29.78%, respectively). The *p*-values were <0.0001. The clearance rate was used to understand the recovery effect of lactate after 10 min of exercise then 20 min of rest. The SPSPE-1X, SPSPE-2X, and SPSPE-5X groups were significantly higher than the vehicle group (by 1.23-fold (*p* < 0.0001), 1.25-fold (*p* < 0.0001), and 1.38-fold (*p* < 0.0001), respectively) and the isocaloric group (by 1.24-fold (*p* = 0.0006), 1.26-fold (*p* < 0.0001), and 1.39-fold (*p* < 0.0001), respectively). The effect of SPSPE supplementation on serum lactate levels was also dose dependent (Table 3).

### 3.5. Effect of SPSPE Supplementation on Fatigue-Related Biochemical Indicators after the 10-min Swim Test or a 90-min Swim Test and 60-min Rest

**Figure 3:**
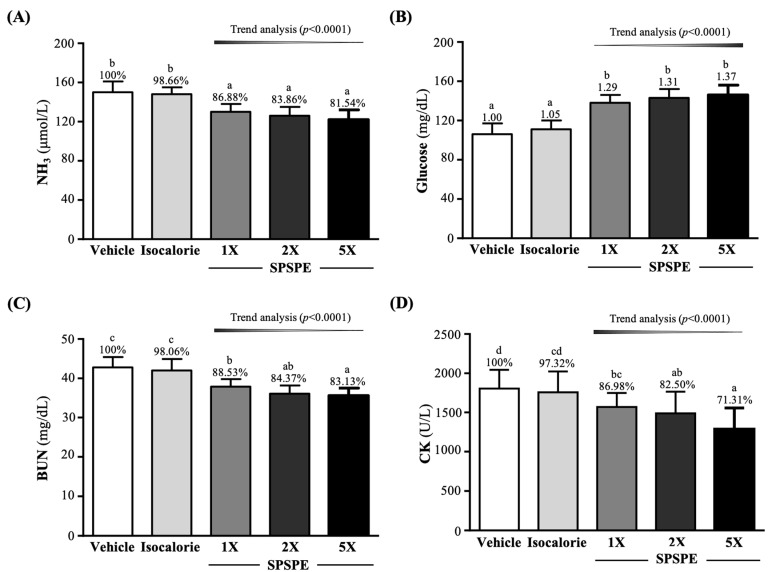
Effect of supplementation with SPSPE on (**A**) NH3, (**B**) glucose, (**C**) BUN, and (**D**) CK. Data are expressed as mean ± SD for *n* = 10 mice per group. The different superscript letters (a, b, c, d) above each bar indicate a significant difference at *p* < 0.05. NH_3_: blood ammonia; BUN: blood urea nitrogen; CK: creatine kinase.

In addition to the lactate level, we also evaluated the NH_3_ and glucose concentration after the 10-min swim test. As shown in Figure 3A, the NH_3_ levels in the SPSPE-1X, SPSPE-2X, and SPSPE-5X groups were significantly lower than the vehicle and isocaloric group, respectively, with *p*-values < 0.0001. The effect of SPSPE supplementation on the serum NH_3_ level was also dose dependent (*p* < 0.0001). The glucose level showed the opposite trend: the SPSPE-1X, SPSPE-2X, and SPSPE-5X groups were significantly greater than the vehicle and isocaloric group, respectively, with *p*-values < 0.0001. The effect of SPSPE supplementation on serum glucose levels was also dose dependent (*p* < 0.0001) (Figure 3B). 

The serum BUN level and the exercise injury index CK were measured 60 min after the 90-min swimming test. As shown in Figure 3C, the BUN levels in the SPSPE-1X, SPSPE-2X, and SPSPE-5X groups were significantly lower than the vehicle and isocaloric group, respectively, with *p*-values < 0.0001. The effect of SPSPE supplementation on the serum BUN level was also dose dependent (*p* < 0.0001). CK activity in the vehicle, isocaloric, SPSPE-1X, SPSPE-2X, and SPSPE-5X groups was 1805 ± 239, 1757 ± 266, 1570 ± 180, 1489 ± 277, and 1287 ± 271 (U/L). Compared with the vehicle group, the SPSPE-1X, SPSPE-2X, and SPSPE-5X groups were significantly reduced by 13.02% (*p* = 0.0441), 17.50% (*p* = 0.0078), and 28.69% (*p* = 0.0001), respectively. In addition, the SPSPE-2X and SPSPE-5X groups were also significantly reduced compared to the isocaloric group by 15.24% (*p* = 0.0227) and 26.73% (*p* = 0.0005), respectively. The effect of SPSPE supplementation on serum CK activity was also dose dependent (*p* < 0.0001) (Figure 3D).

### 3.6. Effect of SPSPE Supplementation on Liver and Muscle Glycogen Levels

**Figure 4:**
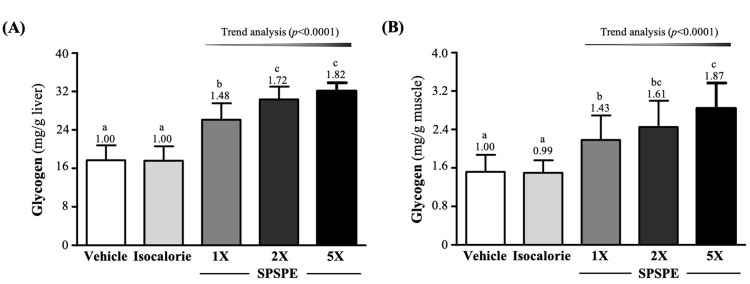
Effect of supplementation with SPSPE on (**A**) liver and (**B**) glucose. Data are expressed as mean ± SD for *n* = 10 mice per group. The different superscript letters (a, b, c) above each bar indicate a significant difference at *p* < 0.05.

The liver glycogen contents of the SPSPE-1X, SPSPE-2X, and SPSPE-5X groups were significantly greater than the vehicle and isocaloric group, respectively, with *p* values < 0.0001 (Figure 4A).As shown in Figure 4B, the muscle glycogen content in the SPSPE-1X, SPSPE-2X, and SPSPE-5X groups was significantly increased compared to the vehicle group (by 1.43-fold (*p* = 0.0022), 1.61-fold (*p* < 0.0001), and 1.87-fold (*p* < 0.0001), respectively) and the isocaloric group (by 1.45-fold (*p* = 0.0018), 1.63-fold (*p* < 0.0001), and 1.89-fold (*p* < 0.0001), respectively). The effect of SPSPE supplementation on the liver and muscle glycogen content was also dose dependent (*p* < 0.0001).

### 3.7. Effect of SPSPE Supplementation on Biochemical Variables at the End of the Experiment

We investigated whether the effects of four-week SPSPE supplementation on improved exercise and anti-fatigue performance were accompanied by biochemical changes in the blood. As shown in Table 4, in each index, no significant difference was observed in for levels/activities between each group (*p* > 0.05). Myoglobin is only found in cardiomyocytes and oxidized skeletal muscle fibers. Myoglobin is released due to tissue injury, which is usually related to the release of enzymes, such as lactate dehydrogenase (LDH), CK, and ALT. It is the earliest marker of myocardial infarction and rhabdomyolysis [20]. Supplementation with SPSPE for 4 consecutive weeks did not cause any damage to the mice’s heart or kidney function.

### 3.8. Effect of SPSPE Supplementation on Tissue Histology

**Figure 5:**
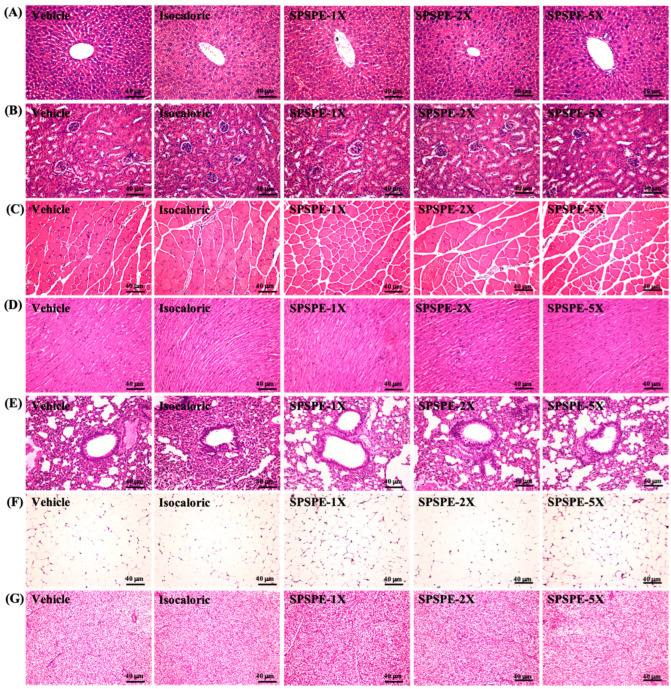
Effect of SPSPE supplementation on (**A**) liver, (**B**) kidney, (**C**) muscle, (**D**) heart, (**E**) lung, (**F**) adipocyte tissue, and (**G**) BAT tissue in mice. H&E stain, magnification: 200×; bar, 40 μm; BAT magnification: 100×; bar, 80 μm.

At the end of the study, we performed histological examination of the liver, kidney, muscle, heart, lung, EFP, and BAT of the mice, and no abnormalities were observed in each group (Figure 5). These results indicate that SPSPE has no adverse effects on organs and tissues at the doses tested in this study.

## 4. Discussion

In recent years, with the change of lifestyle and the gradual development of food technology, increasingly more foods have been concentrated and extracted to form nutritious and convenient nutritional supplements [21]. In this study, supplementation with different doses of SPSPE for 4 consecutive weeks significantly increased the muscle strength, endurance performance, and muscle and glycogen storage of mice. Furthermore, SPSPE also delayed and reduced the production of biochemical indicators of fatigue, such as lactate, NH_3_, CK etc., after exercise, and accelerated fatigue recovery. In addition, through biochemical blood analysis and histopathological observation, we found that SPSPE did cause harm to the health of mice. 

SPSPE is made by extracting whole silver perch according to the proportion of different parts, using a constant-temperature and high-pressure method. Therefore, this supplement is rich in protein and amino acids, mainly composed of BCAA, which is an important nutrient for tissue synthesis, energy supply, and health maintenance [22]. Among them, leucine and isoleucine can be metabolized into acetoacetyl CoA through transamination (TA) [23], enter the citric acid cycle, and generate more energy for working muscles [24]. In addition, isoleucine and valine can be converted into α-keto acid by transamination, metabolized into succinyl-CoA, converted into malic acid and pyruvate, and finally converted into alanine [25]. When BCAA is converted into alanine through the above-mentioned pathways, the body can transport alanine through the blood to the liver, convert it into pyruvate and then into glucose, and then transport it back to the muscles through the blood as an energy source for exercise [26]. Another study showed that the activation of PI3K and p70S6 kinases promoted by leucine can cause a loss of glycogen synthase kinase 3 (GSK-3) activity, which is a proline-directed serine-threonine kinase that inactivates glycogen synthase (GS) and stops GS, thereby promoting and enhancing glycogen synthesis [27,28]. Therefore, BCAAs are beneficial by increasing glycogen storage, although not as directly as sugar-sweetened supplements. This seems to explain the data in this study. After 4 consecutive weeks of supplementation with SPSPE, which is rich in BCAAs, glycogen storage in the liver and muscles increased significantly (Figure 4A,B). The result of a previous study was similar to ours, which found that six weeks of supplementation with BCAA combined with swimming training, compared with a sedentary control group, could significantly increase glycogen storage in the liver and muscles. In addition, a significant improvement in the exercise endurance performance of rats and an extension of the time from exercise to exhaustion were observed [29]. Long-term or strenuous exercise can lead to a decrease in blood sugar, mainly because exercise stimulates the activity of glucose transporters on the muscle fiber cell membrane. However, it increases the glucose uptake of cells for glycolysis, and promotes glycolysis in the liver to increase the blood sugar concentration to provide energy [30]. In addition to BCAA, alanine can also be transported through the blood circulation to the liver, where gluconeogenesis is converted to glucose, which can then be transported through the blood circulation to the muscles for use [31]. A previous study showed that BCAA supplements can delay central nervous system fatigue and improve long-term aerobic endurance performance by increasing the ratio of free tryptophan and reducing the synthesis of serotonin in the brain [32]. Therefore, supplementation with SPSPE for four consecutive weeks can not only increase glycogen storage but also provide energy usage for exercise, thereby improving exercise endurance performance (Figure 2). In addition, a previous study noted that subjects supplemented with 300 mg/kg BCAA for three consecutive days had significantly higher blood glucose concentrations after exercise and recovered 30 min post-exercise compared to the maltodextrin-supplemented placebo group’s stability [30]. This seems to explain the results of our study. After 10 min of unloaded swimming, the SPSPE 1X, 2X, and 5X groups showed a significant increase in blood glucose levels compared to the vehicle and isocaloric groups (Figure 3B).

In addition to increasing the energy required during exercise, the reduction of biochemical parameters of fatigue after exercise is also important. As in our previous studies, blood biochemical indicators, such as lactate, NH_3_, and BUN concentrations, and CK activity, which increase when the length of exercise and intensity are increased and gradually recover during rest, are used as indicators to evaluate exercise fatigue [16,17,33]. Lactate is a product of glycolysis in the anaerobic energy system. With prolongation of exercise, the H^+^ concentration increases and the pH value in blood and muscle tissue decreases, which causes inhibition of glycolysis. In addition, Ca^2+^ related to muscle contraction is released, causing various metabolic and physiological side effects, and resulting in muscle damage and decreased exercise capacity [34]. As exercise progresses, the amount of oxygen taken up and the amount of oxygen delivered to muscle tissue decreases, which regulates the body by preventing pyruvate from effectively entering the TCA cycle and converting it into lactate. This causes lactate to accumulate and peak at the end of exercise. Another study also confirmed that the level of lactic acid supplemented with BCAA immediately after exercise was significantly lower than that of the placebo group, and returned to a stable level 30 min after exercise [35]. This is in line with the trend of our research results (Table 3). Another indicator ammonia increases significantly with exercise intensity or time. The direct source of ammonia production is the purine nucleotide cycle. When the utilization rate of adenosine triphosphate (ATP) exceeds the rate of ATP production, adenosine monophosphate (AMP) is converted to inosine monophosphate (IMP) during ATP resynthesis. At this time, ammonia significantly increases and accumulates in skeletal muscles during high-intensity or long-term exercise [36]. Ammonia is metabolized into BUN via the urea cycle. When the accumulation of ammonia toxicity is too high, this affects the continuous coordination of key areas of the central nervous system [37]. A previous study speculated that during endurance exercise, the increase in the proportion of fat as an energy source was due to the activation of fat oxidation while the relative decrease in the proportion of protein as an energy source reduced the blood ammonia concentration [35]. In our study, we found that supplementation with SPSPE for 4 weeks significantly reduced NH3 and BUN levels after exercise challenges (Figure 3A,C). CK activity indicates muscle damage, which is caused by myocardial infarction, muscular dystrophy, severe muscle breakdown, autoimmune myositis, and acute renal failure [38]. A previous study pointed out that supplementation with 12g of BCAA for 14 consecutive days effectively reduced CK activity and improved endurance exercise-related muscle damage [39]. In our previous study, mice supplemented with soy protein isolate and exercise training significantly reduced the CK activity in the serum after exercise [40]. As a result of this study, after supplementation with SPSPE for 4 consecutive weeks, CK activity was significantly reduced (Figure 3D).

Nutritional supplements or resistance training can increase muscle mass and strength and help reduce muscle damage caused by oxidative stress or inflammatory processes. Forelimb grip strength is a physical test used to assess the overall health of the musculoskeletal system and to evaluate the coordination and adaptability related to sports in neurological research [41,42]. However, there seems to be a subtle relationship between muscle mass and strength. The increase in muscle mass may help increase muscle strength. However, an increase in muscle strength does not necessarily increase muscle mass. In a past study, patients with liver cirrhosis received BCAA treatment for 24 weeks, and found that muscle mass decreased slightly but muscle mass increased significantly [43]. Another study supplemented mice with chicken essence for 4 consecutive weeks, which significantly improved the gripping performance of the forelimbs, and a dose effect was observed after increasing the supplement dose [16]. Although supplementation with SPSPE did not increase muscle mass, this might be due to the supplementation period only being 4 weeks. Thus, longer-term experimental observation is required. However, supplementation still significantly improved the grip strength of the forelimbs (Figure 1).

When evaluating the use of nutritional supplements or special processed foods as medicines or health products, safety is the primary consideration. Fish soup has a long history as a nutritional supplement and is widely used in East and South Asian countries. In recent years, the use of food industry technology to extract fish to produce fish essence can control quality and meet the needs of modern life. At present, among all the related studies on meat essence, chicken essence has been investigated the most while comparatively few studies have investigated fish essence, which may be due to the food culture and breeding cost [44]. In addition, a previous study has pointed out that despite having the same efficacy, there were differences in some bioactive peptides between different meat sources, and different boiling processes also have an impact on the nutritional content [45]. Therefore, no study has investigated the compositional differences of different meat-derived essences. Only in terms of an efficacy comparison, our research confirmed that SPSPE and chicken essence also improved exercise performance and anti-fatigue [15,16]. However, fish protein is easy to digest and is rich in animal-derived protein and limited essential amino acids, and long-chain omega 3 (ω -3) in polyunsaturated fatty acid (PUFA) [46]. It has many health effects, including reducing the risk of heart disease, inflammation, and joint inflammation. At the same time, fish protein is also a rich source of biologically active peptides. If eaten at a reasonable concentration, it can control inflammation (antioxidant peptides) by inhibiting enzymes in the ren-in-angiotensin aldosterone system (RAAS), control blood pressure, and maintain bone health [47]. In this study, SPSPE was supplemented for 4 consecutive weeks, and no abnormal changes in liver function, kidney function, or blood lipid related-indexes were observed in the blood (Table 4). In addition, from a histopathological point of view, it was found that supplementation with SPSPE did not cause pathology or damage to organs (Figure 5). 

## 5. Conclusions

In summary, our results provide evidence that supplementation with SPSPE for four consecutive weeks could significantly increase muscle strength and increase exercise endurance performance by increasing glycogen storage. In addition, supplementation significantly reduced post-exercise biochemical parameters of fatigue, such as the lactate, NH_3_, and BUN concentration and CK activity. Supplementation with the proper amount of SPSPE would not cause damage to various physiological indicators and organs. In addition to being used as a traditional nutritional supplement, fish soup/essence has also been proven to have the benefits of improving exercise performance, delaying the generation of exercise fatigue, and accelerating recovery.

## Figures and Tables

**Table 1:** Nutritional content of the SPSPE supplement.

**Nutrition Facts**	**/100 g SPSPE**
Total calories	376 kcal
Protein	90.8
Fat	0.1
Saturated fat	-
Trans fat	-
Carbohydrate	2.9
Sodium	376 mg
**Hydrolyzed Amino Acid Profiles**	**g/100 g**
Leucine	2.98
Isoleucine	1.33
Valine	2.04
Cystine	1.43
tryptophan	0.11
methionine	1.75
threonine	2.96
Histidine	1.18
Tyrosine	0.89
Alanine	8.45
Glycine	15.54
Serine	2.45
Proline	11.31
Phenylalanine	2.24
Lysine	3.43
Arginine	6.18
Aspartic acid	5.66
Glutamic acid	10.87
Total BCAA (leucine, isoleucine, and valine)	6.35

**Table 2:** Effect of SPSPE supplementation on body weight, body composition, and water and diet intake.

Characteristics	Vehicle	Isocaloric	SPSPE-1X	SPSPE-2X	SPSPE-5X
Initial BW (g)	29.6 ± 0.7 ^a^	29.7 ± 0.7 ^a^	29.8 ± 0.5 ^a^	30.0 ± 0.7 ^a^	30.0 ± 0.6 ^a^
1st week BW	31.3 ± 0.8 ^a^	31.3 ± 1.0 ^a^	31.2 ± 1.0 ^a^	31.4 ± 0.9 ^a^	31.5 ± 1.3 ^a^
2nd week BW	33.7 ± 0.9 ^a^	33.5 ± 0.9 ^a^	33.7 ± 1.2 ^a^	33.5 ± 1.5 ^a^	33.7 ± 1.3 ^a^
3rd week BW	35.2 ± 0.7 ^a^	35.0 ± 1.2 ^a^	35.0 ± 1.2 ^a^	35.1 ± 1.5 ^a^	35.1 ± 1.5 ^a^
4th week BW	36.0 ± 0.7 ^a^	36.1 ± 1.1^a^	36.0 ± 1.6 ^a^	36.1 ± 1.8 ^a^	36.0 ± 1.6 ^a^
5th week BW	37.0 ± 0.7 ^a^	37.0 ± 0.9 ^a^	37.0 ± 1.6 ^a^	37.2 ± 2.0 ^a^	37.0 ± 1.4 ^a^
Final BW (g)	37.5 ± 0.7 ^a^	37.7 ± 0.6 ^a^	37.7 ± 2.0 ^a^	37.8 ± 2.0 ^a^	37.6 ± 1.2 ^a^
Water intake (mL/mouse/day)	7.1 ± 0.3 ^a^	7.1 ± 0.3 ^a^	7.1 ± 0.4 ^a^	7.0 ± 0.2 ^a^	7.0 ± 0.3 ^a^
Diet (g/mouse/day)	6.6 ± 0.8 ^a^	6.6 ± 0.9 ^a^	6.6 ± 1.0 ^a^	6.6 ± 0.8 ^a^	6.6 ± 1.0 ^a^
Calorie intake from diet (Chow 5001) (Kcal/mouse/day) (A)	22.3 ± 2.9 ^a^	22.0 ± 3.0 ^a^	22.3 ± 3.4 ^a^	22.3 ± 2.8 ^a^	22.2 ± 3.3 ^a^
Calorie intake from supplements (Kcal/mouse/day) (B)	0.00	0.13	0.13	0.27	0.66
Total daily calorie intake (Kcal/mouse/day) (A) + (B)	22.3 ± 2.6 ^a^	22.2 ± 2.7 ^a^	22.5 ± 3.1 ^a^	22.6 ± 2.6 ^a^	22.9 ± 3.0 ^a^
Liver (g)	2.17 ± 0.18 ^a^	2.18 ± 0.17 ^a^	2.18 ± 0.14 ^a^	2.18 ± 0.07 ^a^	2.18 ± 0.08 ^a^
Kidney (g)	0.61 ± 0.04 ^a^	0.61 ± 0.07 ^a^	0.61 ± 0.04 ^a^	0.61 ± 0.05 ^a^	0.61 ± 0.07 ^a^
Muscle (g)	0.37 ± 0.02 ^a^	0.37 ± 0.03 ^a^	0.37 ± 0.02 ^a^	0.37 ± 0.02 ^a^	0.37 ± 0.04 ^a^
Heart (g)	0.22 ± 0.04 ^a^	0.21 ± 0.03 ^a^	0.21 ± 0.03 ^a^	0.22 ± 0.02 ^a^	0.21 ± 0.02 ^a^
Lung (g)	0.26 ± 0.03 ^a^	0.27 ± 0.02 ^a^	0.27 ± 0.03 ^a^	0.27 ± 0.03 ^a^	0.26 ± 0.02 ^a^
EFP (g)	0.35 ± 0.06 ^a^	0.35 ± 0.09 ^a^	0.36 ± 0.02 ^a^	0.36 ± 0.02 ^a^	0.35 ± 0.04 ^a^
BAT (g)	0.09 ± 0.02 ^a^	0.09 ± 0.01 ^a^	0.09 ± 0.02 ^a^	0.09 ± 0.01 ^a^	0.09 ± 0.02 ^a^
Relative liver weight (%)	5.79 ± 0.40 ^a^	5.79 ± 0.36 ^a^	5.78 ± 0.24 ^a^	5.78 ± 0.27 ^a^	5.79 ± 0.05 ^a^
Relative kidney weight (%)	1.62 ± 0.08 ^a^	1.63 ± 0.16 ^a^	1.62 ± 0.04 ^a^	1.61 ± 0.05 ^a^	1.62 ± 0.14 ^a^
Relative muscle weight (%)	0.99 ± 0.03 ^a^	0.98 ± 0.07 ^a^	0.97 ± 0.02 ^a^	0.98 ± 0.03 ^a^	0.97 ± 0.09 ^a^
Relative heart weight (%)	0.58 ± 0.09 ^a^	0.57 ± 0.06 ^a^	0.57 ± 0.05 ^a^	0.57 ± 0.03 ^a^	0.56 ± 0.04 ^a^
Relative lung weight (%)	0.69 ± 0.09 ^a^	0.71 ± 0.06 ^a^	0.71 ± 0.05 ^a^	0.70 ± 0.05 ^a^	0.70 ± 0.04 ^a^
Relative EFP weight (%)	0.92 ± 0.13 ^a^	0.93 ± 0.21 ^a^	0.95 ± 0.02 ^a^	0.95 ± 0.03 ^a^	0.93 ± 0.08 ^a^
Relative BAT weight (%)	0.24 ± 0.04 ^a^	0.24 ± 0.03 ^a^	0.23 ± 0.03 ^a^	0.24 ± 0.02 ^a^	0.25 ± 0.04 ^a^

Data are expressed as mean ± SD (*n* = 10 mice per group). The superscript a indicates that there is no significant difference in the same column of data. EFP: epididymal fat pad; BAT: brown adipose tissue.

**Table 3:** Effect of SPSPE supplementation on lactate levels after the 10-min swim.

Time Point	Vehicle	Isocaloric	SPSPE-1X	SPSPE-2X	SPSPE-5X
	Lactate (mmol/L)				
Before swimming (A)	3.34 ± 0.38 ^a^	3.32 ± 0.32 ^a^	3.25 ± 0.37 ^a^	3.45 ± 0.29 ^a^	3.33 ± 0.36 ^a^
After swimming (B)	7.65 ± 0.57 ^c^	7.26 ± 0.50 ^c^	6.18 ± 0.71 ^b^	6.00 ± 0.72 ^ab^	5.54 ± 0.74 ^a^
After a 20 min rest (C)	6.39 ± 0.47 ^c^	6.09 ± 0.54 ^c^	4.92 ± 0.52 ^b^	4.76 ± 0.42 ^b^	4.27 ± 0.52 ^a^
	**Rates of lactate production and clearance**				
Production rate = B/A	2.30 ± 0.10 ^e^	2.19 ± 0.09 ^d^	1.90 ± 0.06 ^c^	1.74 ± 0.08 ^b^	1.66 ± 0.06 ^a^
Clearance rate = (B − C)/B	0.16 ± 0.01 ^a^	0.16 ± 0.03 ^a^	0.20 ± 0.04 ^b^	0.21 ± 0.03 ^b^	0.23 ± 0.04 ^b^

The lactate production rate (B/A) was the value of the lactate level after exercise (B) divided by that before exercise (A). The clearance rate (B−C)/B was defined as the lactate level after swimming (B) minus that after 20 min of rest (C) divided by that after swimming (B). Data are expressed as mean ± SD (*n* = 10 mice per group). Values in the same row with different superscript letters (a, b, c, d, e) differ significantly between groups, *p* < 0.05.

**Table 4:** Effect of SPSPE supplementation on lactate levels after the 10-min swim.

	Vehicle	Isocaloric	SPSPE-1X	SPSPE-2X	SPSPE-5X
AST (U/L)	110 ± 18 ^a^	111 ± 15 ^a^	111 ± 14 ^a^	110 ± 12 ^a^	110 ± 16 ^a^
ALT (U/L)	58 ± 13 ^a^	57 ± 6 ^a^	57 ± 10 ^a^	58 ± 7 ^a^	58 ± 9 ^a^
ALB (mg/dL)	3.17 ± 0.13 ^a^	3.17 ± 0.08 ^a^	3.17 ± 0.12 ^a^	3.17 ± 0.14 ^a^	3.17 ± 0.24 ^a^
BUN (mg/dL)	23.4 ± 3.2 ^a^	23.8 ± 1.1 ^a^	23.7 ± 1.8 ^a^	23.3 ± 1.7 ^a^	23.2 ± 1.3 ^a^
CREA (mg/dL)	0.42 ± 0.04 ^a^	0.41 ± 0.04 ^a^	0.41 ± 0.03 ^a^	0.41 ± 0.03 ^a^	0.42 ± 0.05 ^a^
UA (mg/dL)	1.8 ± 0.9 ^a^	1.7 ± 0.5 ^a^	1.7 ± 0.5 ^a^	1.7 ± 0.3 ^a^	1.8 ± 0.3 ^a^
TP (mg/dL)	5.2 ± 0.5 ^a^	5.3 ± 0.1 ^a^	5.3 ± 0.2 ^a^	5.3 ± 0.3 ^a^	5.2 ± 0.3 ^a^
TC (mg/dL)	130 ± 14 ^a^	130 ± 13 ^a^	129 ± 9 ^a^	130 ± 12 ^a^	128 ± 20 ^a^
TG (mg/dL)	161 ± 16 ^a^	162 ± 22 ^a^	162 ± 25 ^a^	161 ± 11 ^a^	161 ± 28 ^a^
CK (U/L)	275 ± 34 ^a^	276 ± 40 ^a^	281 ± 21 ^a^	278 ± 33 ^a^	281 ± 37 ^a^
Myoglobin (ng/mL)	3.59 ± 0.47 ^a^	3.60 ± 0.82 ^a^	3.57 ± 0.59 ^a^	3.60 ± 0.66 ^a^	3.62 ± 0.46 ^a^

Data are expressed as mean ± SD (*n* = 10 mice per group). The superscript a indicates that there is no significant difference in the same column of data. AST: aspartate aminotransferase; ALT: alanine transaminase; ALB: albumin; BUN: blood urea nitrogen; CREA: creatinine; UA: uric acid; TP: total protein; TC: total cholesterol; TG: triacylglycerol; CK: creatine kinase.

## Data Availability

The data presented in this study are available on request from the corresponding author. The data are not publicly available due to trade secrets and patents.
